# Low-Carbohydrate Diet and Type 2 Diabetes Risk in Japanese Men and Women: The Japan Public Health Center-Based Prospective Study

**DOI:** 10.1371/journal.pone.0118377

**Published:** 2015-02-19

**Authors:** Akiko Nanri, Tetsuya Mizoue, Kayo Kurotani, Atsushi Goto, Shino Oba, Mitsuhiko Noda, Norie Sawada, Shoichiro Tsugane, for the Japan Public Health Center-Based Prospective Study Group

**Affiliations:** 1 Department of Epidemiology and Prevention, Center for Clinical Sciences, National Center for Global Health and Medicine, Tokyo, Japan; 2 Department of Diabetes Research, National Center for Global Health and Medicine, Tokyo, Japan; 3 Department of Health Promotion, National Institute of Public Health, Wako, Japan; 4 Epidemiology and Prevention Group, Research Center for Cancer Prevention and Screening, National Cancer Center, Tokyo, Japan; Indiana University Richard M. Fairbanks School of Public Health, UNITED STATES

## Abstract

**Objective:**

Evidence is sparse and contradictory regarding the association between low-carbohydrate diet score and type 2 diabetes risk, and no prospective study examined the association among Asians, who consume greater amount of carbohydrate. We prospectively investigated the association of low-carbohydrate diet score with type 2 diabetes risk.

**Methods:**

Participants were 27,799 men and 36,875 women aged 45–75 years who participated in the second survey of the Japan Public Health Center-Based Prospective Study and who had no history of diabetes. Dietary intake was ascertained by using a validated food-frequency questionnaire, and low-carbohydrate diet score was calculated from total carbohydrate, fat, and protein intake. The scores for high animal protein and fat or for high plant protein and fat were also calculated. Odds ratios of self-reported, physician-diagnosed type 2 diabetes over 5-year were estimated by using logistic regression.

**Results:**

During the 5-year period, 1191 new cases of type 2 diabetes were self-reported. Low-carbohydrate diet score for high total protein and fat was significantly associated with a decreased risk of type 2 diabetes in women (*P* for trend <0.001); the multivariable-adjusted odds ratio of type 2 diabetes for the highest quintile of the score were 0.63 (95% confidence interval 0.46–0.84), compared with those for the lowest quintile. Additional adjustment for dietary glycemic load attenuated the association (odds ratio 0.75, 95% confidence interval 0.45–1.25). When the score separated for animal and for plant protein and fat, the score for high animal protein and fat was inversely associated with type 2 diabetes in women, whereas the score for high plant protein and fat was not associated in both men and women.

**Discussion:**

Low-carbohydrate diet was associated with decreased risk of type 2 diabetes in Japanese women and this association may be partly attributable to high intake of white rice. The association for animal-based and plant-based low-carbohydrate diet warrants further investigation.

## Introduction

Type 2 diabetes has been increasing worldwide [1]. In Japan, in particular, the prevalence of diabetes has increased from 6.9 to 9.5 million between 1997 and 2012 [2]. In addition, according to the National Health and Nutrition Survey in Japan in most recently, one out of four men and one out of five women have diabetes or pre-diabetes in Japanese adults [2]. However, the prevalence of obesity, a strong determinant of type 2 diabetes, in Japan is much lower compared with those in Western countries [3]. This might be attributed to not only difference of genetic factors but also that of dietary factors between Asian and Western populations. Among Japanese who consume white rice as a staple food, the % energy from carbohydrate is higher but % energy from fat is lower compared with Western population [4,5]. Thus, the association of macronutrient intake with diabetes in Japanese is of great interest.

In a meta-analysis of 22 cohort studies including thirteen US studies, six European studies, and three Asian studies (no Japanese study) [6], carbohydrate intake was associated with an increased risk of type 2 diabetes, whereas protein and fat intake was not associated. However, if one macronutrient intake is high, the others become low. Therefore, carbohydrate, fat, and protein intake should be simultaneously considered as macronutrient balance or overall diet. Halton et al [7] created a simple summary score designated the “low-carbohydrate diet score” according to relative levels of fat, protein, and carbohydrate intake. Previously, two prospective studies have examined the association of the score with type 2 diabetes risk [8,9]. In the Health Professionals Follow-Up Study [8], low-carbohydrate diet score was significantly associated with an increased risk of type 2 diabetes. However, the Nurses’ Health Study observed no association [9]. Moreover, the score for high animal protein and fat was associated with an increased risk of type 2 diabetes in men [8], whereas the score for high vegetable protein and fat was associated with a decreased risk in women [9].

We previously reported an increased risk of type 2 diabetes associated with rice intake [10]. In Japan, rice and processed rice contributes 46.4% of total carbohydrate intake [4]. Given these, we anticipate that low-carbohydrate diet score may be associated with a decreased risk of type 2 diabetes rather than increased risk among Japanese population. Thus, we prospectively examined the association between low-carbohydrate diet score and type 2 diabetes risk in Japanese adults by using data from a large-scale population-based cohort study in Japan. We also examined the association of carbohydrate, fat, and protein intake with type 2 diabetes risk.

## Methods

### Study population

The Japan Public Health Center-based Prospective (JPHC) Study was launched in 1990 for cohort I and in 1993 for cohort II [11]. The participants of cohort I included residents, aged 40 to 59 years, in five Japanese Public Health Center areas (Iwate, Akita, Nagano, Okinawa, and Tokyo); the participants of cohort II included residents, aged 40 to 69 years, in six Public Health Center areas (Ibaraki, Niigata, Kochi, Nagasaki, Okinawa, and Osaka). Although we did not collect written informed consent, study participants were informed of the objectives of the study, and participants who responded to the questionnaire survey were regarded as consenting to participate in the survey. A questionnaire survey was conducted at baseline (first survey), 5-year follow-up (second survey), and 10-year follow-up (third survey). Information on medical histories and health-related lifestyle, smoking, drinking, and dietary habits, was obtained at each survey.

From the study population at first survey (n = 140,420), 113,403 participants responded to the questionnaire at the first survey. Of these, 89,947 participants responded to the second survey, including the diet-related portion. We excluded 14,359 participants who reported a history of type 2 diabetes (n = 6816) or severe disease (n = 8680), including cancer, cerebrovascular disease, myocardial infarction, chronic liver disease, and renal disease at the second survey. Then, we excluded 744 participants who reported extreme total energy intakes (i.e., outside the mean ± 3 standard deviation according to sex). Finally, we excluded 10,170 participants, including participants who died before the third survey and who did not respond to the subsequent third survey, which left a total of 64,674 participants (27,799 men and 36,875 women) in our analysis.

### Ethics statement

This study was approved by the Institutional Review Board of the National Cancer Center of Japan.

### Food frequency questionnaire

At first, second, and third surveys, participants completed a self-administered questionnaire. In the present analysis, data from the second survey was used as baseline survey data, as the questionnaire used for the second survey more comprehensively inquired about food intakes than that used for the first survey. At the second survey, a food frequency questionnaire (FFQ) was used to assess the average intake of 147 food and beverage items over the previous year [12]. For most food items, 9 response options were available to describe consumption frequency, which ranged from rarely (<1 time/month) to ≥7 times/day. A standard portion size was specified for each food, and respondents were asked to denote their usual portion size from 3 options (less than one-half, standard, or >1.5 times). For rice, participants were asked to denote their usual rice-bowl size from three options (small, medium, and large) and the number of bowls consumed daily from 9 options that ranged from <1 to ≥10/day. We calculated the average daily intake of nutrients by multiplying the consumption of each food by its nutrient content per serving and totaling the nutrient intake for all food items. We used the proportion of energy from carbohydrate, fat, and protein (% energy) to calculate low-carbohydrate diet score and to examine the association between each macronutrient intake and type 2 diabetes risk. The validity of the FFQ was assessed subsamples by using either 14- or 28-day dietary records. Spearman’s correlation coefficients between energy-adjusted intake (by residual method) values for carbohydrate, fat, and protein derived from the FFQ and those derived from dietary records were 0.56–0.59, 0.52–0.57, and 0.30, respectively, in men and 0.37–0.39, 0.40–0.46, and 0.27–0.31, respectively, in women [13,14]. With regard to the reproducibility of estimations between the 2 FFQs administered 1 year apart, the respective Spearman’s correlation of coefficients for intake of carbohydrate, fat, and protein were 0.45–0.55, 0.47–0.57, and 0.47–0.57, respectively, in men and 0.41–0.50, 0.38–0.52, and 0.32–0.54, respectively in women [13,15].

Furthermore, we estimated intakes of protein and fat for animal and plant separately; animal food included fish and shellfish, meat and processed meat, egg, milk and dairy products, and butter, and plant food included foods other than animal food. When we assessed the validity and reproducibility of animal or plant protein and fat derived from FFQ, the Spearman’s correlation coefficients between % energy of animal protein and fat and plant protein and fat derived from the FFQ and those derived from dietary records were 0.21, 0.42, 0.59, and 0.39, respectively, in men and 0.26, 0.42, 0.49, and 0.22, respectively, in women. The corresponding values between the 2 FFQs were 0.49, 0.53, 0.60, and 0.64, respectively, in men and 0.48, 0.53, 0.58, and 0.54, respectively, in women.

The low-carbohydrate diet score were developed by Halton et al [7]. Briefly, study participants were divided into 11 categories according to the percentage of energy from carbohydrate, protein, or fat with equal sample size. For carbohydrate, participants in the lowest through highest categories received point from 10 to 0. On the other hand, for protein and fat, participants in the lowest through highest categories received point from 0 to 10. The low-carbohydrate diet score were calculated as total score of carbohydrate, protein, and fat. The maximum score was 30, which represented the highest intake of protein and fat and the lowest intake of carbohydrate. Separate scores were created for animal protein and fat and plant protein and fat.

### Ascertainment of type 2 diabetes

Type 2 diabetes newly diagnosed during the 5-year period after the second survey was determined by a self-administered questionnaire at the third survey. At the third survey, study participants were asked if they had ever been diagnosed with diabetes, and if so, when the initial diagnosis had been made. Because the second survey was used as the starting point of observation for the incidence of type 2 diabetes, only those participants who were diagnosed after 1995 for cohort I and 1998 for cohort II were regarded as incident cases during follow-up. In addition, incident cases included participants who reported using medication for diabetes at the third survey. We did not obtain information on the type of diabetes; however, given that the study participants were 45 years old or over at the beginning of observation, virtually reported cases would be type 2 diabetes. To assess the validity of self-reported diabetes, we examined a series of medical records of some study participants in three districts of the study areas, finding that 94% of self-reported diabetes cases of diabetes were confirmed by medical records [16].

### Statistical analysis

Participants were classified into quintile of low-carbohydrate diet scores for high total, high animal, or high plant protein and fat by sex. Confounding variables considered were as follows: age (year, continuous), study area (11 areas), BMI (<21, 21–22.9, 23–24.9, 25–26.9, or ≥27 kg/m^2^), smoking status (lifetime nonsmoker, former smoker, or current smoker with a consumption of either <20 or ≥20 cigarettes/day), alcohol consumption (nondrinker, occasional drinker, or drinker with a consumption of <150, 150–299, 300–449, or ≥450 g ethanol/week for men and <150 or ≥150 g ethanol/week for women), family history of diabetes mellitus (yes or no), total physical activity (metabolic equivalent task hours/day, quartiles), history of hypertension (yes or no), total energy intake (kcal/day, continuous), and coffee consumption (almost never, <1, 1, or ≥2 cups/day). An indicator variable for missing data was created for each covariate.

Multiple logistic regression analysis was performed to estimate ORs of type 2 diabetes for the quintiles of each low-carbohydrate diet score, taking the lowest category as a reference. The first model was adjusted for age and study area, and the second model was further adjusted for smoking status, alcohol consumption, family history of diabetes mellitus, total physical activity, history of hypertension, total energy intake, and coffee consumption. In the third model, BMI was added to the second model. We assessed quintiles as a continuous variable to evaluate trends after participants were assigned the median value of each score in each quintile. To examine the effect of food groups and nutrients on the association between low-carbohydrate diet score and type 2 diabetes risk, we adjusted for food groups and nutrients one by one. As sensitivity analyses, we repeated the above analyses after excluding women who received hormonal replacement therapy, obese participants (BMI ≥25 kg/m^2^), or older participants (age ≥60 years old).

To examine the association between macronutrient intake and type 2 diabetes risk, we estimated ORs of type 2 diabetes for the quintiles of carbohydrate, fat, and protein intake (% energy) by using multiple logistic regression analysis. The multivariable model was adjusted for age, study area, BMI, smoking status, alcohol consumption, family history of diabetes mellitus, total physical activity, history of hypertension, total energy intake, coffee consumption, magnesium intake (mg/day), calcium intake (mg/day), and vitamin D intake (μg/day). Furthermore, to examine the effect of isocaloric substitution for other macronutrient, we included each macronutrient (% energy) in multivariate nutrient density models. For example, in the model for the association between carbohydrate and type 2 diabetes, we included carbohydrate and protein to examine the effect of substituting carbohydrate for fat, and we included carbohydrate and fat to examine the effect of substituting carbohydrate for protein. In the model for the association between protein and type 2 diabetes, we included protein and fat to examine the effect of substituting protein for carbohydrate, and we included protein and carbohydrate to examine the effect of substituting protein for fat. In the model for the association between fat and type 2 diabetes, we included fat and carbohydrate to examine the effect of substituting fat for protein, and we included fat and protein to examine the effect of substituting fat for carbohydrate.

We analyzed data of cohort I and cohort II combined because the interaction by cohort type on the association between low-carbohydrate diet score and type 2 diabetes risk was not statistically significant. A two-sided *P* <0.05 was regarded as statistically significant. All analyses were performed with Statistical Analysis System (SAS) version 9.3 software (SAS Institute, Cary, NC).

## Results

During the five-year period, 1191 participants were newly diagnosed with diabetes (691 men and 500 women). The characteristics of study participants according to quintile categories of low-carbohydrate diet score for high total, high animal, or high plant protein and fat for men and women, separately are shown in Table 1. Men with a higher score of all low-carbohydrate diet tended to have a higher BMI and a lower prevalence of current smokers than those with a lower score. In addition, men with a higher score of total and plant protein and fat were older than those with a lower score. In women, participants with a higher score of all low-carbohydrate diet were younger than those with a lower score. Among both men and women, all low-carbohydrate diet scores were positively associated with animal fat, plant fat, and animal protein intake but inversely associated with plant protein intake (excluding the score for high plant protein and fat).

**Table 1:** Baseline characteristics of participants according to quintile categories of low-carbohydrate diet score.

	Low-carbohydrate, high total protein and fat score			Low-carbohydrate, high animal protein and fat score			Low-carbohydrate, high plant protein and fat score		
	Q1 (low)	Q3	Q5 (high)	Q1 (low)	Q3	Q5 (high)	Q1 (low)	Q3	Q5 (high)
Men									
No of subjects	5762	6142	5792	5538	5447	5492	6279	5907	6142
Age (years)	56.5 ± 8.0	56.1 ± 7.8	57.4 ± 7.7	56.9 ± 8.1	56.1 ± 7.7	56.9 ± 7.7	56.1 ± 7.8	56.2 ± 7.7	57.3 ± 7.8
Body mass index^1^ (kg/m^2^)	23.3 ± 2.7	23.6 ± 2.8	23.8 ± 2.9	23.3 ± 2.8	23.6 ± 2.8	23.8 ± 2.9	23.4 ± 2.8	23.5 ± 2.8	23.7 ± 2.9
Total physical activity^1^ (MET-hour/week)	34.2 ± 6.8	33.9 ± 6.7	34.0 ± 6.7	34.1 ± 6.8	33.8 ± 6.7	33.9 ± 6.7	34.1 ± 6.9	33.9 ± 6.7	33.8 ± 6.7
Current smoker^1^ (%)	47.7	47.4	42.0	46.7	47.4	44.2	49.5	47.4	40.4
Current drinker^1^ (%)	58.6	74.6	64.2	54.6	74.0	69.4	65.6	72.8	62.5
Coffee consumption^1^ (≥1 cup/day, %)	31.6	34.9	34.4	30.2	34.0	35.1	35.1	32.5	34.0
Family history of diabetes (%)	8.2	8.2	8.1	8.2	8.3	7.9	8.0	8.0	8.5
History of hypertension (%)	15.6	17.5	17.5	15.9	17.4	17.1	15.3	18.3	18.0
Total energy intake (kcal/day)	1920 ± 617	2213 ± 646	2589 ± 902	1942 ± 651	2211 ± 658	2603 ± 897	1993 ± 624	2260 ± 717	2400 ± 825
Carbohydrate intake (% energy/day)	64.7 ± 6.0	51.6 ± 7.7	43.7 ± 6.7	65.0 ± 5.8	52.1 ± 7.0	42.7 ± 6.6	59.7 ± 8.0	51.7 ± 9.7	49.4 ± 7.6
Fat intake (% energy/day)	15.8 ± 3.8	22.7 ± 3.3	32.5 ± 5.5	16.2 ± 4.4	22.4 ± 4.2	32.2 ± 5.9	18.0 ± 5.6	23.3 ± 6.1	28.6 ± 6.3
Animal fat	7.7 ± 2.9	12.6 ± 2.9	20.2 ± 5.7	7.2 ± 2.5	12.3 ± 2.3	21.1 ± 5.2	11.1 ± 5.0	13.6 ± 5.8	14.3 ± 5.6
Plant fat	8.1 ± 2.4	10.1 ± 2.9	12.3 ± 3.5	9.0 ± 3.3	10.1 ± 3.3	11.0 ± 3.2	7.0 ± 1.6	9.8 ± 1.4	14.3 ± 2.9
Protein intake (% energy/day)	11.3 ± 1.4	13.2 ± 1.7	16.7 ± 2.3	11.5 ± 1.7	13.2 ± 2.0	16.3 ± 2.5	11.9 ± 2.1	13.7 ± 2.3	15.3 ± 2.4
Animal protein	4.3 ± 1.4	7.0 ± 1.3	10.8 ± 2.6	4.2 ± 1.3	6.9 ± 1.2	11.0 ± 2.4	6.0 ± 2.4	7.5 ± 2.8	8.0 ± 2.8
Plant protein	7.0 ± 1.0	6.3 ± 1.3	5.9 ± 1.6	7.4 ± 1.1	6.3 ± 1.2	5.3 ± 1.2	5.9 ± 0.9	6.2 ± 1.2	7.3 ± 1.5
Women									
No of subjects	7483	7515	8098	6955	6783	8013	7904	7163	8140
Age (years)	58.2 ± 8.2	56.4 ± 7.8	56.7 ± 7.6	58.2 ± 8.1	56.5 ± 7.8	56.6 ± 7.7	57.9 ± 8.3	56.5 ± 7.9	56.8 ± 7.5
Body mass index^1^ (kg/m^2^)	23.5 ± 3.2	23.4 ± 3.1	23.5 ± 3.1	23.5 ± 3.2	23.4 ± 3.1	23.5 ± 3.1	23.4 ± 3.2	23.4 ± 3.1	23.5 ± 3.1
Total physical activity^1^ (MET-hour/week)	32.7 ± 5.9	32.8 ± 5.7	32.9 ± 5.8	32.8 ± 5.8	32.8 ± 5.6	32.9 ± 5.8	32.6 ± 5.9	32.9 ± 5.7	32.9 ± 5.6
Current smoker^1^ (%)	4.5	4.7	4.5	4.3	5.0	5.0	5.7	4.6	4.3
Current drinker^1^ (%)	9.1	13.8	12.9	7.5	14.2	14.2	12.4	12.5	12.6
Coffee consumption^1^ (≥1 cup/day, %)	31.7	39.3	37.1	29.7	40.0	38.9	37.1	37.3	35.6
Family history of diabetes (%)	7.2	9.9	8.8	7.1	9.7	8.8	8.1	8.8	9.2
History of hypertension (%)	21.1	18.5	17.7	21.7	18.3	17.3	19.6	17.8	18.6
Total energy intake (kcal/day)	1559 ± 540	1905 ± 552	2295 ± 779	1602 ± 586	1880 ± 546	2273 ± 775	1657 ± 594	1931 ± 611	2104 ± 710
Carbohydrate intake (% energy/day)	67.2 ± 4.5	55.7 ± 2.6	44.9 ± 5.4	67.1 ± 4.9	55.9 ± 3.3	44.8 ± 5.7	61.6 ± 8.8	55.7 ± 8.1	51.0 ± 6.1
Fat intake (% energy/day)	18.7 ± 3.9	27.7 ± 2.8	35.9 ± 4.9	19.0 ± 4.4	27.4 ± 3.3	35.9 ± 5.3	22.3 ± 6.7	27.7 ± 6.5	31.7 ± 5.0
Animal fat	8.6 ± 3.1	14.7 ± 3.2	21.8 ± 5.8	7.6 ± 2.5	14.5 ± 2.0	23.0 ± 5.3	13.9 ± 6.4	15.3 ± 6.5	15.0 ± 4.9
Plant fat	10.1 ± 2.9	13.0 ± 3.2	14.1 ± 3.7	11.4 ± 3.8	12.9 ± 3.4	13.0 ± 3.3	8.5 ± 1.8	12.3 ± 1.4	16.7 ± 3.1
Protein intake (% energy/day)	12.4 ± 1.3	15.1 ± 1.4	17.7 ± 2.2	12.5 ± 1.5	15.0 ± 1.6	17.4 ± 2.3	13.6 ± 2.4	15.0 ± 2.1	16.4 ± 2.3
Animal protein	4.9 ± 1.5	8.1 ± 1.5	11.5 ± 2.6	4.5 ± 1.4	8.1 ± 1.2	11.7 ± 2.4	7.4 ± 3.1	8.2 ± 2.8	8.5 ± 2.6
Plant protein	7.5 ± 1.1	7.0 ± 1.2	6.2 ± 1.7	8.0 ± 1.2	7.0 ± 1.1	5.7 ± 1.2	6.2 ± 1.1	6.8 ± 1.3	7.9 ± 1.5

Abbreviations: MET, metabolic equivalent; Q, quintile.

Data are mean ± standard deviation unless otherwise indicated.

^1^ Subjects with missing information were excluded (body mass index: n = 566 in men, n = 894 in women; total physical activity: n = 4663 in men, n = 6442 in women; smoking status: n = 641 in men, n = 2332 in women; alcohol consumption: n = 605 in men, n = 1197 in women; coffee consumption: n = 1451 in men, n = 1880 in women).

ORs and 95% CIs of type 2 diabetes according to low-carbohydrate diet scores for high total, high animal, or high plant protein and fat in men and women are shown in Table 2. The low-carbohydrate diet score for total protein and fat was significantly associated with a decreased risk of type 2 diabetes with adjustment for covariates including BMI in women (*P* for trend = 0.0004); the multivariable-adjusted OR (95% CI) for the highest quintile of the score was 0.63 (0.46–0.84) compared with the lowest quintile. Intake of fish, vegetable, and soy and soy products, which are potentially protective against type 2 diabetes, was higher among participants with a high low-carbohydrate diet score (S1 Table). After additional adjustment for intakes of these foods, the inverse association between the low-carbohydrate diet score and type 2 diabetes remained statistically significant (Table 3). However, adjustment for animal fat intake (which was positively associated with the low-carbohydrate diet score) or dietary glycemic load (which was inversely associated with this score; S1 Table), attenuated the associations, which became statistically not-significant (*P* for trend = 0.055 and 0.17 after additional adjustment for animal fat and dietary glycemic index, respectively; Table 3). In men, the low-carbohydrate diet score was not associated with type 2 diabetes.

**Table 2:** Odds ratios and 95% confidence intervals of type 2 diabetes according to quintile categories of low-carbohydrate diet score.

	Q1 (low)	Q2	Q3	Q4	Q5 (high)	*P* for trend^1^
Men						
Low-carbohydrate, high total protein and fat score						
Median score	3 (0–6)	9 (7–10)	13 (11–15)	18 (16–20)	24 (21–30)	
No of subjects	5762	5227	6142	4876	5792	
No of cases	138	130	164	126	133	
Age- and area-adjusted model^2^	1.00 (reference)	1.05 (0.82, 1.34)	1.13 (0.90, 1.42)	1.10 (0.86, 1.40)	0.97 (0.76, 1.23)	0.87
Multivariable model^3^	1.00 (reference)	1.09 (0.85, 1.40)	1.19 (0.94, 1.50)	1.14 (0.89, 1.47)	1.03 (0.80, 1.34)	0.70
Additionally adjusted for body mass index^4^	1.00 (reference)	1.09 (0.85, 1.40)	1.16 (0.92, 1.48)	1.10 (0.86, 1.42)	1.00 (0.77, 1.30)	0.97
Low-carbohydrate, high animal protein and fat score						
Median score	3 (0–6)	9 (7–11)	14 (12–16)	19 (17–22)	26 (23–30)	
No of subjects	5538	5517	5447	5805	5492	
No of cases	132	137	151	142	129	
Age- and area-adjusted model^2^	1.00 (reference)	1.06 (0.83, 1.35)	1.18 (0.93, 1.50)	1.04 (0.82, 1.32)	0.99 (0.78, 1.27)	0.90
Multivariable model^3^	1.00 (reference)	1.09 (0.85, 1.39)	1.23 (0.96, 1.57)	1.08 (0.84, 1.39)	1.07 (0.82, 1.38)	0.69
Additionally adjusted for body mass index^4^	1.00 (reference)	1.10 (0.86, 1.41)	1.21 (0.95, 1.54)	1.06 (0.83, 1.36)	1.03 (0.80, 1.34)	0.93
Low-carbohydrate, high plant protein and fat score						
Median score	8 (0–9)	10 (10–11)	13 (12–14)	16 (15–17)	20 (18–30)	
No of subjects	6279	4846	5907	4625	6142	
No of cases	173	118	157	110	133	
Age- and area-adjusted model^2^	1.00 (reference)	0.88 (0.69, 1.12)	0.96 (0.77, 1.20)	0.86 (0.67, 1.10)	0.78 (0.61, 0.98)	0.044
Multivariable model^3^	1.00 (reference)	0.90 (0.71, 1.15)	0.98 (0.79, 1.23)	0.89 (0.69, 1.14)	0.80 (0.63, 1.02)	0.084
Additionally adjusted for body mass index^4^	1.00 (reference)	0.91 (0.72, 1.17)	0.99 (0.79, 1.24)	0.88 (0.69, 1.13)	0.79 (0.62, 1.01)	0.065
Women						
Low-carbohydrate, high total protein and fat score						
Median score	5 (0–8)	12 (9–14)	17 (15–19)	21 (20–23)	26 (24–30)	
No of subjects	7483	7817	7515	5962	8098	
No of cases	129	118	89	72	92	
Age- and area-adjusted model^2^	1.00 (reference)	0.90 (0.70, 1.16)	0.71 (0.54, 0.94)	0.72 (0.54, 0.97)	0.67 (0.51, 0.88)	0.0009
Multivariable model^3^	1.00 (reference)	0.91 (0.71, 1.18)	0.70 (0.53, 0.93)	0.71 (0.53, 0.97)	0.62 (0.46, 0.83)	0.0004
Additionally adjusted for body mass index^4^	1.00 (reference)	0.93 (0.72, 1.20)	0.71 (0.54, 0.94)	0.71 (0.53, 0.97)	0.63 (0.46, 0.84)	0.0004
Low-carbohydrate, high animal protein and fat score						
Median score	3 (0–6)	10 (7–13)	16 (14–18)	21 (19–23)	27 (24–30)	
No of subjects	6955	8417	6783	6707	8013	
No of cases	117	118	104	74	87	
Age- and area-adjusted model^2^	1.00 (reference)	0.86 (0.66, 1.11)	0.95 (0.73, 1.24)	0.68 (0.51, 0.91)	0.66 (0.50, 0.87)	0.002
Multivariable model^3^	1.00 (reference)	0.88 (0.68, 1.14)	0.96 (0.73, 1.26)	0.70 (0.52, 0.94)	0.63 (0.47, 0.85)	0.002
Additionally adjusted for body mass index^4^	1.00 (reference)	0.89 (0.69, 1.16)	0.97 (0.74, 1.27)	0.70 (0.52, 0.94)	0.64 (0.48, 0.87)	0.002
Low-carbohydrate, high plant protein and fat score						
Median score	9 (0–11)	13 (12–14)	16 (15–17)	19 (18–20)	23 (21–30)	
No of subjects	7904	6217	7163	7451	8140	
No of cases	119	92	98	94	97	
Age- and area-adjusted model^2^	1.00 (reference)	1.01 (0.77, 1.34)	0.95 (0.72, 1.24)	0.88 (0.67, 1.16)	0.81 (0.62, 1.07)	0.092
Multivariable model^3^	1.00 (reference)	1.04 (0.79, 1.37)	0.97 (0.73, 1.27)	0.89 (0.67, 1.19)	0.81 (0.61, 1.08)	0.091
Additionally adjusted for body mass index^4^	1.00 (reference)	1.05 (0.79, 1.38)	0.98 (0.74, 1.30)	0.91 (0.68, 1.21)	0.81 (0.61, 1.08)	0.10

Abbreviation: Q, quintile.

^1^Based on multiple logistic regression analysis, with the median value of low-carbohydrate diet score assigned to quintile of the diet score.

^2^Adjusted for age (year) and study area (11 areas).

^3^Adjusted for age, study area, smoking status (never, past, current with a consumption of <20 or ≥20 cigarettes/day), alcohol consumption (nondrinker, occasional drinker, or drinker with a consumption of <150, 150–299, 300–449, or ≥450 g ethanol/week), family history of diabetes mellitus (yes or no), total physical activity (quartile, metabolic equivalent-hour/day), history of hypertension (yes or no), total energy intake (kcal/day), and coffee consumption (almost never, <1, 1, or ≥2 cups/day).

^4^Additionally adjusted for body mass index (<21, 21–22.9, 23–24.9, 25–26.9, or ≥27 kg/m^2^).

**Table 3:** Odds ratios and 95% confidence intervals of type 2 diabetes according to quintile categories of low-carbohydrate diet score after adjustment for foods and nutrients in women.

	Q1 (low)	Q2	Q3	Q4	Q5 (high)	*P* for trend^1^
Multivariable model^2^	1.00 (reference)	0.93 (0.72, 1.20)	0.71 (0.54, 0.94)	0.71 (0.53, 0.97)	0.63 (0.46, 0.84)	0.0004
Adjusted for cereal^3^	1.00 (reference)	0.98 (0.75, 1.27)	0.77 (0.57, 1.04)	0.79 (0.57, 1.11)	0.73 (0.51, 1.06)	0.048
Adjusted for potatoes^3^	1.00 (reference)	0.92 (0.71, 1.20)	0.71 (0.53, 0.94)	0.71 (0.53, 0.97)	0.63 (0.47, 0.84)	0.0004
Adjusted for legumes^3^	1.00 (reference)	0.92 (0.71, 1.19)	0.70 (0.53, 0.93)	0.70 (0.52, 0.95)	0.61 (0.45, 0.83)	0.0003
Adjusted for nuts^3^	1.00 (reference)	0.92 (0.71, 1.19)	0.71 (0.53, 0.94)	0.71 (0.52, 0.96)	0.62 (0.46, 0.84)	0.0004
Adjusted for vegetable^3^	1.00 (reference)	0.92 (0.71, 1.19)	0.70 (0.53, 0.93)	0.71 (0.52, 0.96)	0.62 (0.46, 0.84)	0.0004
Adjusted for fruit^3^	1.00 (reference)	0.93 (0.72, 1.20)	0.71 (0.54, 0.94)	0.72 (0.53, 0.97)	0.63 (0.47, 0.85)	0.0007
Adjusted for fish^3^	1.00 (reference)	0.89 (0.69, 1.16)	0.67 (0.50, 0.90)	0.67 (0.48, 0.92)	0.57 (0.41, 0.79)	0.0002
Adjusted for meat^3^	1.00 (reference)	0.93 (0.72, 1.21)	0.71 (0.54, 0.95)	0.72 (0.52, 0.99)	0.64 (0.46, 0.89)	0.002
Adjusted for egg^3^	1.00 (reference)	0.92 (0.71, 1.19)	0.70 (0.53, 0.93)	0.71 (0.52, 0.96)	0.62 (0.46, 0.84)	0.0004
Adjusted for milk^3^	1.00 (reference)	0.95 (0.73, 1.23)	0.74 (0.55, 0.98)	0.74 (0.55, 1.01)	0.66 (0.49, 0.90)	0.003
Adjusted for dietary GI^3^	1.00 (reference)	0.94 (0.72, 1.21)	0.72 (0.54, 0.96)	0.73 (0.53, 0.99)	0.65 (0.47, 0.89)	0.002
Adjusted for dietary GL^3^	1.00 (reference)	0.98 (0.73, 1.31)	0.78 (0.55, 1.10)	0.81 (0.53, 1.22)	0.75 (0.45, 1.25)	0.17
Adjusted for animal fat^3^	1.00 (reference)	0.96 (0.73, 1.25)	0.75 (0.55, 1.02)	0.77 (0.54, 1.10)	0.71 (0.46, 1.08)	0.055
Adjusted for plant fat^3^	1.00 (reference)	0.93 (0.72, 1.21)	0.71 (0.53, 0.95)	0.72 (0.53, 0.99)	0.63 (0.46, 0.86)	0.001
Adjusted for animal protein^3^	1.00 (reference)	0.93 (0.71, 1.23)	0.72 (0.52, 0.998)	0.73 (0.50, 1.06)	0.64 (0.41, 1.02)	0.031
Adjusted for plant protein^3^	1.00 (reference)	0.93 (0.72, 1.21)	0.72 (0.54, 0.96)	0.73 (0.54, 0.995)	0.66 (0.48, 0.89)	0.002

^1^Based on multiple logistic regression analysis, with the median value of low-carbohydrate diet score assigned to quintile of the diet score.

^2^Adjusted for age (year), study area (11 areas), body mass index (<21, 21–22.9, 23–24.9, 25–26.9, or ≥27 kg/m^2^), smoking status (never, past, current with a consumption of <20 or ≥20 cigarettes/day), alcohol consumption (nondrinker, occasional drinker, or drinker with a consumption of <150, 150–299, 300–449, or ≥450 g ethanol/week), family history of diabetes mellitus (yes or no), total physical activity (quartile, metabolic equivalent-hour/day), history of hypertension (yes or no), total energy intake (kcal/day), and coffee consumption (almost never, <1, 1, or ≥2 cups/day).

^3^Additionally adjusted for intake of each food or nutrient (continuous).

When separate scores were created for animal protein and fat and plant protein and fat, the low-carbohydrate diet score for high animal protein and fat was inversely associated with type 2 diabetes risk in women (*P* for trend = 0.002) (Table 2). Regarding the score for high plant protein and fat, although the OR of type 2 diabetes tended to decrease with increasing this score in both men and women, the association was not statistically significant (*P* for trend = 0.07 in men and 0.09 in women; Table 2).

In a sensitivity analysis among non-obese women (n = 26,072), the multivariable-adjusted ORs (95% CI) for the highest versus lowest quintile of each score were 0.61 (0.39–0.95) for the low-carbohydrate diet score (*P* for trend = 0.013), 0.61 (0.38–0.96) for the score for animal protein and fat (*P* for trend = 0.032), and 0.71 (0.47–1.09) for the score for plant protein and fat (*P* for trend = 0.075). Among women aged <60 years old (n = 23,057), the corresponding values were 0.53 (0.35–0.80) for the low-carbohydrate diet score (*P* for trend = 0.0009), 0.58 (0.39–0.88) for the score for animal protein and fat (*P* for trend = 0.006), and 0.74 (0.49–1.11) for the score for plant protein and fat (*P* for trend = 0.076). When analysis was limited to women who did not receive hormonal replacement therapy (n = 34,024), the results did not materially change (data not shown). In a subgroup of non-smoking and non-drinking men (n = 4731), the multivariable-adjusted ORs (95% CI) for the highest versus lowest quintile of the low-carbohydrate diet score was 1.25 (0.68–2.30).

The association between macronutrient intake and type 2 diabetes are shown in S2 Table in men and S3 Table in women. In men, plant fat intake was associated with a decreased risk of type 2 diabetes after additional adjustment for any macronutrient (carbohydrate, protein, or animal fat). In women, carbohydrate intake was significantly and positively associated with type 2 diabetes risk after adjustment for covariates including protein intake. In contrast, fat intake was significantly and inversely associated with type 2 diabetes risk after adjustment for covariates including protein intake. Carbohydrate and protein intake in men and protein intake in women was not associated with type 2 diabetes risk.

## Discussion

In this large-scale, population-based, prospective study in Japanese adults, low-carbohydrate diet score was associated with a decreased risk of type 2 diabetes in women. This association was attenuated after further adjustment for glycemic load. In analyses considering the source of protein and fat, the score for animal protein and fat was inversely associated with type 2 diabetes risk in women but not in men. The score for plant protein and fat was not associated with type 2 diabetes risk in both men and women. To our knowledge, this is the first prospective study to examine the association of low-carbohydrate diet score with type 2 diabetes in Asia.

As expected, low-carbohydrate diet score for high total protein and fat was associated with a decreased risk of type 2 diabetes in women. However, this finding disagrees with those in previous studies. The Nurses’ Health Study [9] and the Health Professionals Follow-Up Study [8] have observed that the diet score was significantly associated with increased risk of type 2 diabetes after adjustment for covariates other than BMI. Although the significant associations were attenuated after additional adjustment for BMI, the positive association remained statistical significant in the latter study [8]. Multivariate nutrient density model [17], another method examining the balance of carbohydrate, protein, and fat intake, includes one macronutrient (% energy) as the exposure and total energy intake and other energy-yielding nutrients (% energy), except the nutrient to be replaced, as confounders. The coefficients from the model can be interpreted as isocaloric substitution of units of % energy from the other macronutrient for the units of % energy from one macronutrient. Previously, four prospective Western studies have examined the association between substitution of macronutrients and type 2 diabetes risk using this method [18–21]. Replacement of fat with carbohydrate was associated with a decreased risk of type 2 diabetes in the Alpha-Tocopherol, Beta-Carotene Cancer Prevention Study among Finnish male smokers aged 50 to 69 [20], but not in the European Prospective Investigation into Cancer and Nutrition (EPIC)-Potsdam Study among German men and women aged 35 to 65 [19]. Regarding replacing protein with carbohydrate, the Alpha-Tocopherol, Beta-Carotene Cancer Prevention and EPIC-Potsdam Studies have reported a decreased risk of type 2 diabetes [19,20]. In addition, replacing carbohydrate with protein was associated with an increased risk of type 2 diabetes in the EPIC-Netherlands Study among Dutch men and women aged 21 to70 [21] and the Malmö Diet and Cancer cohort among Swedish men and women aged 45 to75 [18]. Our findings of an inverse association between low-carbohydrate diet score and type 2 diabetes risk also disagree with these findings.

The differences of finding between the present and the previous Western studies might partly be explained by the difference of intake and major sources of carbohydrate and fat between Japanese and Western population. Carbohydrate intake was higher in Japanese than Western population. Means of carbohydrate intake in the lowest and highest quintile of low-carbohydrate diet score were 64.7 and 43.7% energy, respectively, among men and 67.2 and 44.9% energy, respectively, among women in the present study. In contrast, the corresponding values were 57.7 and 37.3% energy, respectively, in the Health Professionals Follow-Up Study [8]. In addition, the major sources of carbohydrate are rice and processed rice (46.4%) in Japan [4]. In the US, they are soft drinks and soda (13.9%), yeast breads and rolls (10.0%), and cakes, cookies, quick bread pastry, and pie (8.3%), and rice and cooked grains contribute to only 3.1% of total carbohydrate intake [22]. White rice, a high glycemic index food, was associated with a significant increased risk of type 2 diabetes, especially in Asians including Japanese [23]. Moreover, we previously reported the positive association between dietary glycemic load, which represents both the quantity and quality of the carbohydrate consumed, and type 2 diabetes risk in the same study population [24]. The inverse association between low-carbohydrate diet score and type 2 diabetes risk was attenuated after adjustment for dietary glycemic load in the present study, suggesting that the observed association with low-carbohydrate diet score might be partly ascribed to dietary glycemic load. Furthermore, adjustment with animal fat, which was positively associated with low-carbohydrate diet score, attenuated the inverse association between this score and type 2 diabetes risk, suggesting that the inverse association might also be explained by animal fat. Among Japanese population, fish intake is much higher while meat intake is lower, compared with Western population. This difference in major dietary source of animal food intake might explain the inconsistent finding between the present and Western studies. Alternatively, genetic factors might partly account for the inconsistent findings. In Japanese population, who has lower insulin secretary capacity than Western [25], glucose may be more easily increased after carbohydrate intake.

In the present study, the decreased risk of type 2 diabetes associated with low-carbohydrate diet score was observed in women but not in men. Although the reason for no association in men is unclear, the proportion of smoker and drinker was much higher in men than in women. Strong impact of the effect of residual confounding due to these lifestyles may have obscured the association in men. Similar to the present findings, we observed an increased risk of type 2 diabetes associated with rice intake and dietary glycemic load in women but not in men in the same study population [10,24].

When the low-carbohydrate diet score was separated for animal and for plant protein and fat, the score for plant protein and fat was not associated with type 2 diabetes, though the risk of type 2 diabetes for the highest of the score was 19–21% lower compared with the lowest. In the Nurses’ Health Study [9], low-carbohydrate, high vegetable protein and fat score was associated with a decreased risk of type 2 diabetes. In the Health Professionals Follow-Up Study [8], the score was not associated with type 2 diabetes risk overall subjects, but an inverse association was observed in men younger than age 65. The diet score is expected to be associated with decreased risk of type 2 diabetes by the protective effects of vegetable and polyunsaturated fatty acids from plant foods. Some studies reported a decreased risk of type 2 diabetes associated with high vegetable fat intake [26,27]. In a meta-analysis including seven prospective cohort studies [28], intake of alpha-linolenic acid, which is abundant in plant, has been associated with modestly lower risk of type 2 diabetes. Additionally, linoleic acid intake has been associated with decreased prevalence of impaired glucose metabolism [29]. In the present study, since a small protective association of the low-carbohydrate diet for plant protein and fat with type 2 diabetes cannot be excluded, the association warrants further investigation.

As regards low-carbohydrate diet score for animal protein and fat, results of the two previous US studies are contradictory; one observed an increased risk of type 2 diabetes [8], but another did not [9]. In the present study, low-carbohydrate diet score for animal protein and fat was associated with a decreased, rather than increased, risk of type 2 diabetes in women, but not in men. Our finding of no increased risk of type 2 diabetes associated with low-carbohydrate diet score might be ascribed to lower intake of meat and processed meat among Japanese than Western population. In a study of American men [8], the positive association of low-carbohydrate diet score for high animal protein and fat with type 2 diabetes risk have been found to be mainly due to intake of red and processed meat. The reason for the *decreased* risk of type 2 diabetes associated with low-carbohydrate diet score in the present study might be partly explained by a larger contribution of fish to animal protein and fat intake in Japanese. Higher intakes of fish and n-3 polyunsaturated fatty acid intake have been associated with decreased risk of type 2 diabetes in Asia [30]. Additionally, higher levels of odd-chain saturated fatty acids [31] and vitamin D [32], nutrients in fish, have been associated with decreased risk of type 2 diabetes. Given the inconsistent and limited evidence on the association between low-carbohydrate diet score for animal protein and fat and type 2 diabetes, however, further investigation is warranted.

Major strengths of the present study included our large sample size, the population-based prospective design, the use of a validated FFQ, and adjustment for or stratification by potentially important confounding variables. Further, more gathering data from a Japanese population allowed us to assess any potential association at relatively extremely high amounts of carbohydrate. However, several limitations to the present study warrant mention. First, the diagnosis of type 2 diabetes was ascertained by self-report. However, a validation study conducted in our study population showed fairly good agreement between self-reported diabetes and diabetes as documented in medical records (94%), and the sensitivity of self-reported diabetes was reasonably high (83%). Second, dietary intakes were measured only once and thus may not have reflected long-term intake. This probably has led to an underestimation of the association. Repeated assessment of diet over a long period of time before disease onset will likely provide a better estimate of exposure status. Moreover, given low validity of carbohydrate, fat, and protein intake assessed by FFQ, particularly in women, misclassification for low-carbohydrate diet score may lead to attenuation of the association between this diet score and type 2 diabetes risk. Nevertheless, we observed a statistically significant association between them in women. Third, as shown in S1 Table, intake of some nutrients including fish, vegetables, and soy and soy products was higher among participants with a higher score of low-carbohydrate diet for total protein and fat, and these nutrients could be confounders. However, we observed a significant inverse association between low-carbohydrate diet score and type 2 diabetes risk even after adjustment for these food intakes. Fourth, although the present study is prospective design, reverse causation is a concern because of short follow-up period (5 years). Some patients might already have pre-diabetes or metabolic syndrome at baseline and thus changed their dietary behavior. Because we did not conduct blood glucose examination at baseline among all participants, we were unable to exclude participants with undiagnosed diabetes or pre-diabetes. However, in sensitivity analysis after exclusion of obese or older participants who have high prevalence of these symptoms, we observed similar inverse association between low-carbohydrate diet score and type 2 diabetes risk. Fifth, we excluded participants who died before third survey, which could potentially introduce selection bias. Low-carbohydrate diet has been associated with increased risk of death in Western populations [33], whereas it was associated with decreased risk of death in Japanese [34]. Given this inconsistency, it is difficult to predict the direction of potential bias due to selective participation into the follow-up survey across low-carbohydrate diet score. Finally, we cannot rule out the possibility of unmeasured and residual confounding including socioeconomic status. Additionally, the present study included a sizable number of missing data for covariates, especially physical activity, and this might have influenced the results to some extent.

In conclusion, we observed that low-carbohydrate diet score was significantly associated with a decreased risk of type 2 diabetes in women. This association was attenuated after further adjustment for glycemic load, suggesting a significant contribution of high intake of white rice (a major source of refined carbohydrate) in Japanese. In addition, although the score for plant protein and fat was not associated with type 2 diabetes, a small protective effect of the diet cannot be excluded. The present findings suggest that low-carbohydrate and high-fat and protein diet is protectively associated with type 2 diabetes in Japanese women. The role of plant-based and animal-based low-carbohydrate diet warrants further investigation.

## Supporting Information

S1 Table Energy-adjusted intake (by residual method) of food groups according to quintile categories of low-carbohydrate diet score. (DOCX)

S2 Table Odds ratios and 95% confidence intervals of type 2 diabetes according to quintile categories of intake of carbohydrate, fat, and protein in men. (DOCX)

S3 Table Odds ratios and 95% confidence intervals of type 2 diabetes according to quintile categories of intake of carbohydrate, fat, and protein in women. (DOCX)
