# Supplementary material for: Low-Carbohydrate Diet and Type 2 Diabetes Risk in Japanese Men and Women: The Japan Public Health Center-Based Prospective Study
Source: PLoS One. 2015 Feb 19;10(2):e0118377. doi: 10.1371/journal.pone.0118377 (PMC4335023; doi:10.1371/journal.pone.0118377)
Supplement: S1 Table — (DOCX) [file pone.0118377.s001.docx]

**S1 Table. Energy-adjusted intake^a^ (by residual method) of food groups according to quintile categories of low-carbohydrate diet score**

|  | **Low-carbohydrate, high total protein and fat score** | | |  | **Low-carbohydrate, high animal protein and fat score** | | |  | **Low-carbohydrate, high plant protein and fat score** | | |
| --- | --- | --- | --- | --- | --- | --- | --- | --- | --- | --- | --- |
|  | **Q1 (low)** | **Q3** | **Q5 (high)** |  | **Q1 (low)** | **Q3** | **Q5 (high)** |  | **Q1 (low)** | **Q3** | **Q5 (high)** |
| **Men** |  |  |  |  |  |  |  |  |  |  |  |
| Cereal (g/day) | 745.7 ± 1.8 | 583.2 ± 1.7 | 480.4 ± 1.7 |  | 747.3 ± 1.8 | 587.2 ± 1.8 | 474.6 ± 1.8 |  | 682.8 ± 1.9 | 592.0 ± 2.0 | 536.8 ± 1.9 |
| Potatoes (g/day) | 21.7 ± 0.3 | 22.7 ± 0.3 | 23.0 ± 0.3 |  | 24.3 ± 0.3 | 22.7 ± 0.3 | 20.2 ± 0.3 |  | 16.8 ± 0.3 | 22.1 ± 0.3 | 29.3 ± 0.3 |
| Sugar (g/day) | 3.0 ± 0.1 | 2.7 ± 0.0 | 2.4 ± 0.1 |  | 2.8 ± 0.1 | 2.8 ± 0.1 | 2.4 ± 0.1 |  | 3.2 ± 0.0 | 2.6 ± 0.0 | 2.6 ± 0.0 |
| Legumes (g/day) | 71.8 ± 0.9 | 84.2 ± 0.9 | 102.9 ± 0.9 |  | 84.7 ± 1.0 | 88.1 ± 1.0 | 81.2 ± 1.0 |  | 52.3 ± 0.8 | 79.2 ± 0.8 | 138.4 ± 0.8 |
| Nuts (g/day) | 1.7 ± 0.1 | 2.1 ± 0.1 | 2.4 ± 0.1 |  | 2.2 ± 0.1 | 2.1 ± 0.1 | 1.9 ± 0.1 |  | 1.2 ± 0.1 | 1.8 ± 0.1 | 3.8 ± 0.1 |
| Vegetables (g/day) | 174.3 ± 1.6 | 198.0 ± 1.6 | 206.9 ± 1.6 |  | 194.2 ± 1.7 | 198.2 ± 1.7 | 182.8 ± 1.7 |  | 137.6 ± 1.5 | 188.9 ± 1.5 | 264.7 ± 1.5 |
| Fruit (g/day) | 199.8 ± 2.0 | 175.2 ± 1.9 | 158.3 ± 2.0 |  | 209.5 ± 2.0 | 175.2 ± 2.0 | 145.6 ± 2.0 |  | 172.5 ± 1.9 | 170.1 ± 1.9 | 194.9 ± 1.9 |
| Mushroom (g/day) | 7.0 ± 0.1 | 8.6 ± 0.1 | 9.4 ± 0.1 |  | 7.9 ± 0.1 | 8.7 ± 0.1 | 8.2 ± 0.1 |  | 5.5 ± 0.1 | 8.3 ± 0.1 | 11.7 ± 0.1 |
| Seaweed (g/day) | 8.5 ± 0.2 | 9.5 ± 0.1 | 11.0 ± 0.2 |  | 9.4 ± 0.2 | 9.8 ± 0.2 | 9.5 ± 0.2 |  | 6.6 ± 0.1 | 9.5 ± 0.1 | 13.8 ± 0.1 |
| Fish and shellfish (g/day) | 58.4 ± 0.7 | 87.8 ± 0.6 | 124.4 ± 0.7 |  | 58.6 ± 0.7 | 88.8 ± 0.7 | 121.4 ± 0.7 |  | 72.7 ± 0.7 | 92.8 ± 0.7 | 101.9 ± 0.7 |
| Meat and processed meat (g/day) | 41.5 ± 0.5 | 61.3 ± 0.5 | 90.9 ± 0.5 |  | 38.2 ± 0.5 | 59.7 ± 0.5 | 94.5 ± 0.5 |  | 54.2 ± 0.5 | 66.3 ± 0.5 | 67.0 ± 0.5 |
| Egg (g/day) | 23.6 ± 0.4 | 31.5 ± 0.4 | 41.3 ± 0.4 |  | 22.4 ± 0.4 | 31.1 ± 0.4 | 42.7 ± 0.4 |  | 30.3 ± 0.4 | 31.9 ± 0.4 | 31.1 ± 0.4 |
| Milk and milk product (g/day) | 113.6 ± 2.5 | 159.0 ± 2.4 | 215.9 ± 2.5 |  | 100.6 ± 2.5 | 155.1 ± 2.5 | 233.7 ± 2.5 |  | 191.3 ± 2.4 | 152.7 ± 2.5 | 144.3 ± 2.4 |
| Oil and fat (g/day) | 8.1 ± 0.1 | 11.3 ± 0.1 | 14.4 ± 0.1 |  | 8.8 ± 0.1 | 11.2 ± 0.1 | 13.5 ± 0.1 |  | 7.6 ± 0.1 | 11.1 ± 0.1 | 15.3 ± 0.1 |
| Confectioneries (g/day) | 16.9 ± 0.3 | 14.7 ± 0.3 | 13.6 ± 0.3 |  | 18.5 ± 0.3 | 14.3 ± 0.3 | 11.9 ± 0.3 |  | 12.7 ± 0.3 | 14.5 ± 0.3 | 18.1 ± 0.3 |
| Alcohol beverage (g/day) | 387.2 ± 8.8 | 396.9 ± 8.5 | 172.5 ± 8.8 |  | 343.0 ± 9.0 | 409.8 ± 9.1 | 219.2 ± 9.1 |  | 453.6 ± 8.5 | 352.5 ± 8.7 | 208.4 ± 8.6 |
| Non-alcohol beverage (g/day) | 934.5 ± 7.8 | 825.4 ± 7.5 | 744.4 ± 7.8 |  | 966.1 ± 8.0 | 831.6 ± 8.0 | 703.9 ± 8.0 |  | 825.9 ± 7.6 | 825.6 ± 7.7 | 872.6 ± 7.6 |
| Condiment (g/day) | 6.0 ± 0.1 | 8.2 ± 0.1 | 9.7 ± 0.1 |  | 6.6 ± 0.1 | 8.2 ± 0.1 | 8.8 ± 0.1 |  | 5.3 ± 0.1 | 7.9 ± 0.1 | 11.0 ± 0.1 |
| Dietary glycemic index^b^ | 64.7 ± 0.1 | 62.2 ± 0.1 | 59.1 ± 0.1 |  | 64.3 ± 0.1 | 62.2 ± 0.1 | 59.5 ± 0.1 |  | 64.5 ± 0.1 | 62.1 ± 0.1 | 59.6 ± 0.1 |
| Dietary glycemic load | 205.2 ± 0.3 | 163.3 ± 0.3 | 135.8 ± 0.3 |  | 204.9 ± 0.3 | 164.7 ± 0.3 | 134.4 ± 0.3 |  | 191.6 ± 0.4 | 163.9 ± 0.4 | 151.1 ± 0.4 |

| **Women** |  |  |  |  |  |  |  |  |  |  |  |
| --- | --- | --- | --- | --- | --- | --- | --- | --- | --- | --- | --- |
| Cereal (g/day) | 600.2 ± 1.2 | 490.3 ± 1.2 | 390.0 ± 1.2 |  | 596.5 ± 1.3 | 490.8 ± 1.3 | 395.0 ± 1.2 |  | 548.7 ± 1.4 | 496.2 ± 1.4 | 436.1 ± 1.3 |
| Potatoes (g/day) | 31.2 ± 0.3 | 31.6 ± 0.3 | 26.9 ± 0.3 |  | 34.0 ± 0.3 | 31.1 ± 0.3 | 24.9 ± 0.3 |  | 24.3 ± 0.3 | 30.7 ± 0.3 | 35.0 ± 0.3 |
| Sugar (g/day) | 3.1 ± 0.0 | 3.0 ± 0.0 | 2.3 ± 0.0 |  | 2.8 ± 0.0 | 3.1 ± 0.0 | 2.3 ± 0.0 |  | 3.3 ± 0.0 | 2.8 ± 0.0 | 2.5 ± 0.0 |
| Legumes (g/day) | 72.4 ± 0.8 | 85.9 ± 0.8 | 99.9 ± 0.8 |  | 89.3 ± 0.9 | 85.9 ± 0.9 | 79.3 ± 0.8 |  | 50.8 ± 0.7 | 76.2 ± 0.8 | 140.2 ± 0.7 |
| Nuts (g/day) | 1.6 ± 0.1 | 1.9 ± 0.1 | 1.9 ± 0.1 |  | 2.1 ± 0.1 | 1.8 ± 0.1 | 1.5 ± 0.1 |  | 1.0 ± 0.1 | 1.6 ± 0.1 | 3.1 ± 0.1 |
| Vegetables (g/day) | 214.1 ± 1.5 | 234.7 ± 1.5 | 217.9 ± 1.5 |  | 240.9 ± 1.6 | 235.3 ± 1.6 | 198.9 ± 1.5 |  | 162.8 ± 1.4 | 225.4 ± 1.5 | 287.3 ± 1.4 |
| Fruit (g/day) | 298.9 ± 1.9 | 240.1 ± 1.9 | 182.6 ± 1.8 |  | 301.1 ± 2.0 | 240.3 ± 2.0 | 177.3 ± 1.9 |  | 256.8 ± 1.9 | 244.2 ± 2.0 | 221.8 ± 1.9 |
| Mushroom (g/day) | 10.0 ± 0.1 | 11.6 ± 0.1 | 11.1 ± 0.1 |  | 11.2 ± 0.2 | 11.8 ± 0.2 | 9.9 ± 0.1 |  | 8.0 ± 0.1 | 11.0 ± 0.1 | 14.3 ± 0.1 |
| Seaweed (g/day) | 10.4 ± 0.1 | 11.9 ± 0.1 | 12.0 ± 0.1 |  | 11.8 ± 0.1 | 12.0 ± 0.1 | 10.7 ± 0.1 |  | 8.4 ± 0.1 | 11.3 ± 0.1 | 15.2 ± 0.1 |
| Fish and shellfish (g/day) | 59.3 ± 0.5 | 87.5 ± 0.5 | 108.9 ± 0.5 |  | 58.1 ± 0.5 | 88.8 ± 0.5 | 106.7 ± 0.5 |  | 76.0 ± 0.5 | 85.8 ± 0.6 | 92.7 ± 0.5 |
| Meat and processed meat (g/day) | 36.9 ± 0.4 | 54.1 ± 0.4 | 75.7 ± 0.4 |  | 32.0 ± 0.4 | 53.9 ± 0.4 | 80.4 ± 0.4 |  | 51.1 ± 0.4 | 57.8 ± 0.4 | 52.8 ± 0.4 |
| Egg (g/day) | 22.1 ± 0.3 | 28.4 ± 0.3 | 33.1 ± 0.3 |  | 19.9 ± 0.3 | 29.3 ± 0.3 | 33.9 ± 0.3 |  | 30.9 ± 0.3 | 27.6 ± 0.3 | 26.3 ± 0.3 |
| Milk and milk product (g/day) | 150.1 ± 2.4 | 209.0 ± 2.3 | 239.0 ± 2.3 |  | 121.4 ± 2.4 | 205.7 ± 2.4 | 260.3 ± 2.2 |  | 274.3 ± 2.3 | 186.9 ± 2.4 | 163.5 ± 2.2 |
| Oil and fat (g/day) | 9.3 ± 0.1 | 12.5 ± 0.1 | 13.8 ± 0.1 |  | 10.1 ± 0.1 | 12.4 ± 0.1 | 13.2 ± 0.1 |  | 8.3 ± 0.1 | 12.1 ± 0.1 | 15.0 ± 0.0 |
| Confectioneries (g/day) | 26.5 ± 0.3 | 21.4 ± 0.3 | 15.1 ± 0.3 |  | 27.4 ± 0.3 | 21.1 ± 0.3 | 14.3 ± 0.3 |  | 20.1 ± 0.3 | 22.2 ± 0.3 | 20.3 ± 0.3 |
| Alcohol beverage (g/day) | 21.8 ± 1.4 | 25.0 ± 1.4 | 20.1 ± 1.3 |  | 16.0 ± 1.4 | 29.4 ± 1.4 | 25.6 ± 1.3 |  | 36.2 ± 1.3 | 22.9 ± 1.4 | 19.1 ± 1.3 |
| Non-alcohol beverage (g/day) | 885.5 ± 6.8 | 823.0 ± 6.7 | 727.8 ± 6.5 |  | 910.5 ± 7.1 | 832.0 ± 7.1 | 702.0 ± 6.6 |  | 792.6 ± 6.7 | 813.8 ± 6.9 | 845.5 ± 6.6 |
| Condiment (g/day) | 7.3 ± 0.1 | 9.5 ± 0.1 | 9.8 ± 0.1 |  | 8.0 ± 0.1 | 9.4 ± 0.1 | 9.2 ± 0.1 |  | 6.2 ± 0.1 | 9.0 ± 0.1 | 11.3 ± 0.1 |
| Dietary glycemic index^b^ | 63.7 ± 0.1 | 60.4 ± 0.1 | 57.5 ± 0.1 |  | 63.3 ± 0.1 | 60.5 ± 0.1 | 57.9 ± 0.1 |  | 62.7 ± 0.1 | 60.6 ± 0.1 | 58.5 ± 0.1 |
| Dietary glycemic load | 174.6 ± 0.2 | 144.8 ± 0.2 | 116.4 ± 0.2 |  | 173.8 ± 0.2 | 145.0 ± 0.2 | 117.5 ± 0.2 |  | 160.6 ± 0.3 | 145.9 ± 0.3 | 130.3 ± 0.3 |

Abbreviation: Q, quintile.

Data are mean ± standard error.

^a^Adjusted for age (y) and study area (11 areas).

^b^Crude intake
