# Supplementary material for: Low-Carbohydrate Diet and Type 2 Diabetes Risk in Japanese Men and Women: The Japan Public Health Center-Based Prospective Study
Source: PLoS One. 2015 Feb 19;10(2):e0118377. doi: 10.1371/journal.pone.0118377 (PMC4335023; doi:10.1371/journal.pone.0118377)
Supplement: S2 Table — (DOCX) [file pone.0118377.s002.docx]

**S2 Table. Odds ratios and 95% confidence intervals of type 2 diabetes according to quintile categories of intake of carbohydrate, fat, and protein in men**

|  | **Q1 (low)** | **Q2** | **Q3** | **Q4** | **Q5 (high)** | ***P* for trend^a^** |
| --- | --- | --- | --- | --- | --- | --- |
| No of subjects | 5559 | 5560 | 5560 | 5560 | 5560 |  |
| Carbohydrate |  |  |  |  |  |  |
| Median (range) (% of energy) | 40.2 (8.9-44.8) | 48.0 (44.8-50.7) | 53.1 (50.7-55.5) | 58.2 (55.5-61.1) | 65.3 (61.1-87.6) |  |
| No of cases | 122 | 147 | 143 | 143 | 136 |  |
| Multivariable model^b^ + protein^c^ | 1.00 (reference) | 1.26 (0.96, 1.65) | 1.22 (0.90, 1.65) | 1.24 (0.88, 1.74) | 1.19 (0.79, 1.78) | 0.47 |
| Multivariable model^b^ + fat^c^ | 1.00 (reference) | 1.24 (0.93, 1.64) | 1.18 (0.84, 1.65) | 1.18 (0.79, 1.76) | 1.11 (0.66, 1.86) | 0.74 |
| Fat |  |  |  |  |  |  |
| Median (range) (% of energy) | 14.4 (1.4-17.2) | 19.2 (17.2-21.1) | 22.8 (21.1-24.6) | 26.4 (24.6-28.8) | 32.4 (28.8-74.4) |  |
| No of cases | 131 | 147 | 137 | 148 | 128 |  |
| Multivariable model^b^ + protein^c^ | 1.00 (reference) | 1.13 (0.87, 1.46) | 1.04 (0.78, 1.37) | 1.13 (0.83, 1.53) | 0.94 (0.65, 1.37) | 0.71 |
| Multivariable model^b^ + carbohydrate^c^ | 1.00 (reference) | 1.13 (0.87, 1.46) | 1.03 (0.76, 1.39) | 1.11 (0.79, 1.56) | 0.90 (0.57, 1.43) | 0.73 |
| Animal fat |  |  |  |  |  |  |
| Median (range) (% of energy) | 6.6 (0.0-8.5) | 9.9 (8.5-11.1) | 12.4 (11.1-13.8) | 15.3 (13.8-17.2) | 20.3 (17.2-60.9) |  |
| No of cases | 117 | 156 | 140 | 131 | 147 |  |
| Multivariable model^b^ + protein and plant fat^c^ | 1.00 (reference) | 1.47 (1.14, 1.90) | 1.33 (1.00, 1.76) | 1.27 (0.93, 1.73) | 1.46 (1.01, 2.11) | 0.20 |
| Multivariable model^b^ + carbohydrate and plant fat^c^ | 1.00 (reference) | 1.46 (1.13, 1.90) | 1.32 (0.99, 1.75) | 1.24 (0.90, 1.72) | 1.40 (0.92, 2.12) | 0.34 |
| Multivariable model^b^ + protein and carbohydrate^c^ | 1.00 (reference) | 1.46 (1.12, 1.90) | 1.32 (0.99, 1.77) | 1.27 (0.91, 1.77) | 1.51 (0.99, 2.31) | 0.21 |
| Plant fat |  |  |  |  |  |  |
| Median (range) (% of energy) | 6.2 (0.9-7.4) | 8.3 (7.4-9.0) | 9.8 (9.0-10.6) | 11.4 (10.6-12.6) | 14.2 (12.6-55.1) |  |
| No of cases | 148 | 151 | 133 | 144 | 115 |  |
| Multivariable model^b^ + protein and animal fat^c^ | 1.00 (reference) | 0.97 (0.76, 1.24) | 0.86 (0.66, 1.12) | 0.91 (0.69, 1.20) | 0.71 (0.52, 0.97) | 0.030 |
| Multivariable model^b^ + carbohydrate and animal fat^c^ | 1.00 (reference) | 0.98 (0.77, 1.25) | 0.86 (0.66, 1.13) | 0.91 (0.69, 1.21) | 0.71 (0.50, 0.996) | 0.054 |
| Multivariable model^b^ + protein and carbohydrate^c^ | 1.00 (reference) | 0.97 (0.76, 1.24) | 0.85 (0.65, 1.11) | 0.89 (0.67, 1.18) | 0.68 (0.48, 0.94) | 0.021 |
| Protein |  |  |  |  |  |  |
| Median (range) (% of energy) | 10.4 (3.2-11.5) | 12.2 (11.5-12.8) | 13.4 (12.8-14.1) | 14.7 (14.1-15.5) | 16.8 (15.5-38.2) |  |
| No of cases | 128 | 134 | 148 | 156 | 125 |  |
| Multivariable model^b^ + fat^c^ | 1.00 (reference) | 1.14 (0.86, 1.50) | 1.38 (1.01, 1.87) | 1.54 (1.09, 2.19) | 1.36 (0.87, 2.14) | 0.09 |
| Multivariable model^b^ + carbohydrate^c^ | 1.00 (reference) | 1.11 (0.84, 1.45) | 1.32 (0.98, 1.77) | 1.45 (1.03, 2.04) | 1.25 (0.80, 1.96) | 0.16 |
| Animal protein |  |  |  |  |  |  |
| Median (range) (% of energy) | 3.9 (0.0-4.9) | 5.6 (4.9-6.3) | 7.0 (6.3-7.6) | 8.4 (7.6-9.3) | 10.8 (9.3-34.0) |  |
| No of cases | 121 | 150 | 143 | 145 | 132 |  |
| Multivariable model^b^ + fat and plant protein^c^ | 1.00 (reference) | 1.36 (1.04, 1.78) | 1.39 (1.02, 1.89) | 1.47 (1.03, 2.11) | 1.48 (0.93, 2.36) | 0.16 |
| Multivariable model^b^ + carbohydrate and plant protein^c^ | 1.00 (reference) | 1.32 (1.01, 1.71) | 1.32 (0.98, 1.78) | 1.38 (0.98, 1.94) | 1.35 (0.86, 2.12) | 0.26 |
| Multivariable model^b^ + fat and carbohydrate^c^ | 1.00 (reference) | 1.36 (1.04, 1.78) | 1.39 (1.03, 1.87) | 1.47 (1.04, 2.07) | 1.46 (0.94, 2.27) | 0.15 |
| Plant protein |  |  |  |  |  |  |
| Median (range) (% of energy) | 4.7 (0.9-5.3) | 5.7 (5.3-6.0) | 6.3 (6.0-6.6) | 6.9 (6.6-7.3) | 7.9 (7.3-21.7) |  |
| No of cases | 132 | 156 | 144 | 131 | 128 |  |
| Multivariable model^b^ + fat and animal protein^c^ | 1.00 (reference) | 1.17 (0.90, 1.52) | 1.08 (0.80, 1.45) | 1.00 (0.71, 1.41) | 0.94 (0.63, 1.42) | 0.54 |
| Multivariable model^b^ + carbohydrate and animal protein^c^ | 1.00 (reference) | 1.18 (0.90, 1.53) | 1.09 (0.80, 1.47) | 1.01 (0.72, 1.42) | 0.95 (0.63, 1.43) | 0.55 |
| Multivariable model^b^ + fat and carbohydrate^c^ | 1.00 (reference) | 1.17 (0.90, 1.52) | 1.06 (0.79, 1.43) | 0.97 (0.70, 1.35) | 0.88 (0.60, 1.30) | 0.29 |

Abbreviation: Q, quintile.

^a^Based on multiple logistic regression analysis, with the median intake of carbohydrate, fat, and protein assigned to the quintile categories of each intake.

^b^Adjusted for age (year), study area (11 areas), body mass index (<21, 21-22.9, 23-24.9, 25-26.9, or ≥27 kg/m^2^), smoking status (never, past, current with a consumption of <20 or ≥20 cigarettes/day), alcohol consumption (nondrinker, occasional drinker, or drinker with a consumption of <150 or ≥150 g ethanol/week), family history of diabetes mellitus (yes or no), total physical activity (quartile, metabolic equivalent-hour/day), history of hypertension (yes or no), total energy intake (kcal/day), coffee consumption (almost never, <1, 1, or ≥2 cups/day), magnesium intake (mg/day), calcium intake (mg/day), and vitamin D intake (μg/day).

^c^Additionally adjusted for each macronutrient intake (% energy).
