# Supplementary material for: Low-Carbohydrate Diet and Type 2 Diabetes Risk in Japanese Men and Women: The Japan Public Health Center-Based Prospective Study
Source: PLoS One. 2015 Feb 19;10(2):e0118377. doi: 10.1371/journal.pone.0118377 (PMC4335023; doi:10.1371/journal.pone.0118377)
Supplement: S3 Table — (DOCX) [file pone.0118377.s003.docx]

**S3 Table. Odds ratios and 95% confidence intervals of type 2 diabetes according to quintile categories of intake of carbohydrate, fat, and protein in women**

|  | **Q1 (low)** | **Q2** | **Q3** | **Q4** | **Q5 (high)** | ***P* for trend^a^** |
| --- | --- | --- | --- | --- | --- | --- |
| No of subjects | 7375 | 7375 | 7375 | 7375 | 7375 |  |
| Carbohydrate |  |  |  |  |  |  |
| Median (range) (% of energy) | 45.0 (5.5-49.1) | 51.9 (49.1-54.1) | 56.1 (54.1-58.1) | 60.3 (58.1-62.9) | 66.5 (62.9-88.5) |  |
| No of cases | 77 | 89 | 96 | 110 | 128 |  |
| Multivariable model^b^ + protein^c^ | 1.00 (reference) | 1.24 (0.89, 1.73) | 1.33 (0.94, 1.90) | 1.52 (1.03, 2.23) | 1.65 (1.04, 2.60) | 0.027 |
| Multivariable model^b^ + fat^c^ | 1.00 (reference) | 1.15 (0.80, 1.66) | 1.19 (0.78, 1.81) | 1.30 (0.80, 2.12) | 1.32 (0.70, 2.48) | 0.38 |
| Fat |  |  |  |  |  |  |
| Median (range) (% of energy) | 18.9 (2.2-21.8) | 23.9 (21.8-25.6) | 27.2 (25.6-28.9) | 30.7 (28.9-32.9) | 36.1 (32.9-67.5) |  |
| No of cases | 130 | 108 | 90 | 95 | 77 |  |
| Multivariable model^b^ + protein^c^ | 1.00 (reference) | 0.90 (0.68, 1.19) | 0.77 (0.56, 1.05) | 0.85 (0.61, 1.19) | 0.64 (0.43, 0.96) | 0.044 |
| Multivariable model^b^ + carbohydrate^c^ | 1.00 (reference) | 1.00 (0.73, 1.37) | 0.90 (0.61, 1.33) | 1.06 (0.67, 1.69) | 0.91 (0.48, 1.72) | 0.90 |
| Animal fat |  |  |  |  |  |  |
| Median (range) (% of energy) | 7.9 (0.0-10.0) | 11.6 (10.0-13.0) | 14.3 (13.0-15.7) | 17.2 (15.7-19.2) | 22.3 (19.2-56.0) |  |
| No of cases | 126 | 102 | 104 | 88 | 80 |  |
| Multivariable model^b^ + protein and plant fat^c^ | 1.00 (reference) | 0.91 (0.69, 1.21) | 1.00 (0.73, 1.35) | 0.86 (0.61, 1.22) | 0.75 (0.49, 1.15) | 0.20 |
| Multivariable model^b^ + carbohydrate and plant fat^c^ | 1.00 (reference) | 0.98 (0.72, 1.33) | 1.12 (0.79, 1.61) | 1.03 (0.66, 1.59) | 1.01 (0.55, 1.87) | 0.91 |
| Multivariable model^b^ + protein and carbohydrate^c^ | 1.00 (reference) | 1.00 (0.74, 1.35) | 1.16 (0.82, 1.63) | 1.08 (0.72, 1.61) | 1.10 (0.64, 1.88) | 0.69 |
| Plant fat |  |  |  |  |  |  |
| Median (range) (% of energy) | 8.4 (0.4-9.8) | 10.7 (9.8-11.5) | 12.3 (11.5-13.1) | 14.0 (13.1-15.1) | 16.8 (15.1-49.4) |  |
| No of cases | 112 | 116 | 90 | 90 | 92 |  |
| Multivariable model^b^ + protein and animal fat^c^ | 1.00 (reference) | 1.12 (0.85, 1.48) | 0.85 (0.63, 1.15) | 0.88 (0.64, 1.20) | 0.84 (0.60, 1.18) | 0.15 |
| Multivariable model^b^ + carbohydrate and animal fat^c^ | 1.00 (reference) | 1.21 (0.91, 1.62) | 0.97 (0.69, 1.35) | 1.05 (0.72, 1.53) | 1.14 (0.71, 1.83) | 0.82 |
| Multivariable model^b^ + protein and carbohydrate^c^ | 1.00 (reference) | 1.18 (0.89, 1.55) | 0.92 (0.68, 1.26) | 0.98 (0.70, 1.37) | 1.02 (0.70, 1.48) | 0.77 |
| Protein |  |  |  |  |  |  |
| Median (range) (% of energy) | 12.2 (4.8-13.1) | 13.8 (13.1-14.3) | 14.9 (14.3-15.5) | 16.1 (15.5-16.9) | 18.0 (16.9-48.6) |  |
| No of cases | 103 | 112 | 96 | 101 | 88 |  |
| Multivariable model^b^ + fat^c^ | 1.00 (reference) | 1.17 (0.87, 1.58) | 1.04 (0.75, 1.46) | 1.06 (0.74, 1.53) | 0.87 (0.55, 1.38) | 0.46 |
| Multivariable model^b^ + carbohydrate^c^ | 1.00 (reference) | 1.21 (0.89, 1.63) | 1.10 (0.77, 1.55) | 1.15 (0.77, 1.69) | 0.98 (0.60, 1.63) | 0.85 |
| Animal protein |  |  |  |  |  |  |
| Median (range) (% of energy) | 4.7 (0.0-5.7) | 6.6 (5.7-7.3) | 7.9 (7.3-8.6) | 9.4 (8.6-10.3) | 11.7 (10.3-47.9) |  |
| No of cases | 116 | 103 | 99 | 88 | 94 |  |
| Multivariable model^b^ + fat and plant protein^c^ | 1.00 (reference) | 1.09 (0.81, 1.48) | 1.16 (0.82, 1.65) | 1.10 (0.74, 1.64) | 1.24 (0.74, 2.05) | 0.48 |
| Multivariable model^b^ + carbohydrate and plant protein^c^ | 1.00 (reference) | 1.15 (0.84, 1.57) | 1.27 (0.88, 1.82) | 1.24 (0.81, 1.90) | 1.49 (0.85, 2.59) | 0.19 |
| Multivariable model^b^ + fat and carbohydrate^c^ | 1.00 (reference) | 1.12 (0.83, 1.51) | 1.21 (0.86, 1.71) | 1.17 (0.78, 1.74) | 1.37 (0.82, 2.28) | 0.27 |
| Plant protein |  |  |  |  |  |  |
| Median (range) (% of energy) | 5.3 (0.6-5.9) | 6.2 (5.9-6.6) | 6.8 (6.6-7.2) | 7.5 (7.2-7.9) | 8.6 (7.9-25.8) |  |
| No of cases | 90 | 91 | 88 | 108 | 123 |  |
| Multivariable model^b^ + fat and animal protein^c^ | 1.00 (reference) | 0.90 (0.65, 1.23) | 0.78 (0.55, 1.10) | 0.86 (0.60, 1.25) | 0.86 (0.56, 1.33) | 0.61 |
| Multivariable model^b^ + carbohydrate and animal protein^c^ | 1.00 (reference) | 0.90 (0.66, 1.24) | 0.79 (0.56, 1.11) | 0.89 (0.61, 1.29) | 0.91 (0.59, 1.41) | 0.81 |
| Multivariable model^b^ + fat and carbohydrate^c^ | 1.00 (reference) | 0.90 (0.66, 1.23) | 0.79 (0.56, 1.10) | 0.89 (0.62, 1.26) | 0.91 (0.61, 1.35) | 0.79 |

Abbreviation: Q, quintile.

^a^Based on multiple logistic regression analysis, with the median intake of carbohydrate, fat, and protein assigned to the quintile categories of each intake.

^b^Adjusted for age (year), study area (11 areas), body mass index (<21, 21-22.9, 23-24.9, 25-26.9, or ≥27 kg/m^2^), smoking status (never, past, current with a consumption of <20 or ≥20 cigarettes/day), alcohol consumption (nondrinker, occasional drinker, or drinker with a consumption of <150 or ≥150 g ethanol/week), family history of diabetes mellitus (yes or no), total physical activity (quartile, metabolic equivalent-hour/day), history of hypertension (yes or no), total energy intake (kcal/d), coffee consumption (almost never, <1, 1, or ≥2 cups/day), magnesium intake (mg/day), calcium intake (mg/day), and vitamin D intake (μg/day).

^c^Additionally adjusted for each macronutrient intake (% energy).
